# High-Risk Prostate Cancer Treated with Radiation Therapy: Favorable Outcomes in Men with PSA > 20 as the Sole High-Risk Factor

**DOI:** 10.3390/jcm15031119

**Published:** 2026-01-30

**Authors:** Aoi Shimomura, Abed R. Kawakibi, Muzamil Arshad, Stanley L. Liauw

**Affiliations:** 1 Department of Radiation Oncology, Cedars Sinai Medical Center, Los Angeles, CA 90048, USA; 2 Department of Radiation and Cellular Oncology, University of Chicago Medical Center, Chicago, IL 60637, USA

**Keywords:** high-risk prostate cancer, PSA, ADT

## Abstract

**Background/Objectives:** The National Comprehensive Cancer Network (NCCN) classifies prostate cancer with PSA > 20 ng/mL as high risk; however, outcomes within this group are heterogeneous. Emerging data suggest that men with PSA > 20 ng/mL as the sole high-risk feature may have more favorable disease biology. We evaluated outcomes of men with prostate cancer treated with definitive radiation therapy (RT), focusing on the prognostic significance of individual high-risk factors. **Methods:** We analyzed 742 men with prostatic adenocarcinoma treated with curative-intent RT between 2005 and 2021, including 282 meeting traditional NCCN high-risk criteria. Treatment consisted of dose-escalated RT (median 78 Gy), with androgen deprivation therapy (ADT) administered to 94% (median duration 28 months). Primary endpoints were freedom from biochemical failure (FFBF) and distant metastasis (FFDM). Outcomes were assessed using Kaplan–Meier methods and Cox proportional hazards modeling. **Results:** At 5 years, high-risk patients demonstrated FFBF of 83% and FFDM of 89%, with significantly worse outcomes among very high-risk subgroups. Men with PSA > 20 ng/mL as their only high-risk feature (n = 49) achieved superior outcomes compared with other high-risk patients (5-year FFBF 94% vs. 74%; FFDM 97% vs. 82%; both *p* = 0.05), comparable to intermediate-risk disease. On multivariable analysis, Gleason score and clinical T-stage independently predicted poorer outcomes, whereas PSA > 20 alone did not. **Conclusions:** PSA > 20 ng/mL as an isolated high-risk feature is associated with favorable outcomes following definitive RT and appears to be the weakest NCCN high-risk criterion. These findings support refined risk stratification and raise the possibility of treatment de-escalation in select patients.

## 1. Introduction

The traditional National Comprehensive Cancer Network (NCCN) three-tier classification system for prostate cancer includes low, intermediate, and high-risk categories [1]. Since the original risk stratification by D’Amico et al. [2], efforts have been made to refine these broad categories. The introduction of subgroups such as favorable and unfavorable intermediate risk [3] and the distinction between high and very high risk [4] have been a result of the wide variability in prognosis among patients within the same broad risk category. This highlights the importance of proper risk stratification to provide personalized treatment.

When specifically looking at high-risk prostate cancer, studies have shown that men who have high-risk disease based on having Prostate Specific Antigen (PSA) > 20 ng/mL alone have improved disease outcomes and may be more similar to outcomes of men with intermediate-risk disease [5,6,7]. Despite these findings, it remains the standard of care to offer all men with high-risk prostate cancer to receive long-term (18–36 months) androgen deprivation therapy (ADT) with radiation therapy (RT). This recommendation is based on randomized trials [8,9,10] that were largely conducted prior to the contemporary era of dose-escalated, image-guided RT [11]. Notably, long-term ADT can be associated with a range of adverse effects that can significantly impact quality of life [12]. In addition, there may be limited improvement in disease outcomes past 18 months of ADT use and an increased risk of toxicity that could negatively influence non-cancer mortality [13].

The purpose of this study was to explore clinical factors associated with disease outcomes for men with non-metastatic high-risk prostate cancer. Specifically, we sought to evaluate outcomes in a cohort of men treated with primary RT and explore which high-risk factors correlated with treatment failure, to offer further insight on potential avenues to personalize therapy.

## 2. Materials and Methods

This analysis included 742 men with adenocarcinoma of the prostate treated with primary external beam RT with curative intent at the University of Chicago between 2005 and 2021. This study was approved by the University of Chicago Institutional Review Board (IRB #14934A), and patients provided informed consent for inclusion in the registry. Consecutively treated patients meeting eligibility criteria were included in this retrospective analysis. Patients were excluded if they had evidence of metastatic disease at diagnosis, prior definitive local therapy such as surgery or radiation therapy, brachytherapy as monotherapy [14], or incomplete baseline clinical data. Using NCCN risk classification [15], 282 men had high-risk disease (HR), defined as the presence of any one of the following: prostate-specific antigen (PSA) > 20 ng/mL, clinical stage ≥ cT3a, or biopsy Gleason score ≥ 8. A subgroup of men with NCCN very high-risk disease (VHR) had ≥2 high-risk factors, clinical T3b or higher disease, and primary Gleason pattern 5. Patient characteristics are summarized in Table 1.

Patients with evidence of distant metastasis at presentation or who had received alternative primary therapies were excluded. All patients underwent CT-based radiation planning. RT was delivered with curative intent using intensity-modulated radiation therapy (IMRT). The prostate and proximal seminal vesicles were included in the treatment volume; pelvic lymph nodes were included based on risk assessment and physician discretion and treated to a median dose of 50.4 Gy. RT was delivered in daily fractions of 1.8 to 2.0 Gy, with a median total dose of 78 Gy (range, 14.4–160.4 Gy). Details regarding radiation planning have been reported previously [16].

ADT was administered at the discretion of the treating physician and consisted primarily of a gonadotropin-releasing hormone (GnRH) analog, with or without an anti-androgen for dual blockade during the neoadjuvant and concurrent portion of treatment with RT. In general, hormonal therapy duration was chosen based on risk classification, consisting of no ADT for low-risk disease, up to 6 months for intermediate-risk disease, and 28 months for high-risk disease. However, deviations from this paradigm could occur after shared decision-making, factoring in medical comorbidity and patient preference. ADT was typically initiated 2 months before RT and continued concurrently during RT with adjuvant ADT. The duration of ADT was recorded, with a median duration of 28 months (range, 0–209 months).

The primary endpoints were freedom from biochemical failure (FFBF) and freedom from distant metastasis (FFDM). FFBF was defined using the RTOG-ASTRO Phoenix Consensus Conference definition (nadir PSA + 2 ng/mL) [17]. FFDM was defined as the time from the start of RT to the date of radiographically confirmed distant metastasis. FFBF and FFDM were evaluated using the Kaplan-Meier method. Patients were censored at the date of last known follow-up if no event had occurred. Salvage therapies were not considered events; FFBF and FFDM endpoints were driven only by biochemical failure or distant progression by imaging. Imaging was obtained in response to abnormal clinical or lab findings and not at a routine surveillance interval. Imaging modality varied by treatment era and physician discretion; modern molecular imaging such as PSMA PET was not routinely available during most of the study period, and metastatic recurrence was largely defined by CT and bone scan.

Univariate analysis was conducted using the log-rank test to evaluate associations between prognostic factors and clinical outcomes. Multivariable analysis (MVA) was performed using the Cox proportional hazards model, including covariates T stage, PSA, Gleason score, the number of NCCN high-risk factors, and NCCN vHR disease. A two-sided *p*-value of <0.05 was considered statistically significant. Given the exploratory nature of this retrospective analysis, adjustments for multiple comparisons were not performed, and results should be interpreted as hypothesis-generating. All statistical analyses were performed using JMP Pro 15 (Cary, NC, USA).

## 3. Results

### 3.1. Patient and Treatment Characteristics

A total of 282 men with NCCN high-risk prostate cancer were analyzed (Table 1). The median age at treatment was 68 years, and the median pretreatment prostate-specific antigen (PSA) was 19 ng/mL. Most patients (94%) received androgen-deprivation therapy (ADT) for a median of 28 months, and 57% received pelvic nodal radiotherapy.

Clinical T stage at diagnosis was Tx 1%, T1–T2a 43%, T2b–T2c 15%, and T3–T4 41%. Forty-nine men (17%) had PSA > 20 ng/mL as their only high-risk feature, whereas 233 (83%) had other criteria for high-risk disease. The subgroups were similar in age (median 67 vs. 69 years, *p* = 0.51), but men with high-risk disease by PSA > 20 only were more likely to be Black (*p* = 0.006) and have lower Gleason scores (*p* < 0.0001, by nature of not having Gleason 8 disease by definition) and also were treated with less nodal radiation (41% vs. 61%, *p* < 0.01) and ADT (84% vs. 96%, *p* = 0.03). A minority of men had PSA > 100 (8%, n = 23); the proportion of men with PSA > 100 was not significantly different between the two groups (4% for PSA > 20 only and 9% otherwise, *p* = 0.22).

### 3.2. Freedom from Biochemical Failure (FFBF) and Freedom from Distant Metastasis (FFDM)

**Figure 1:**
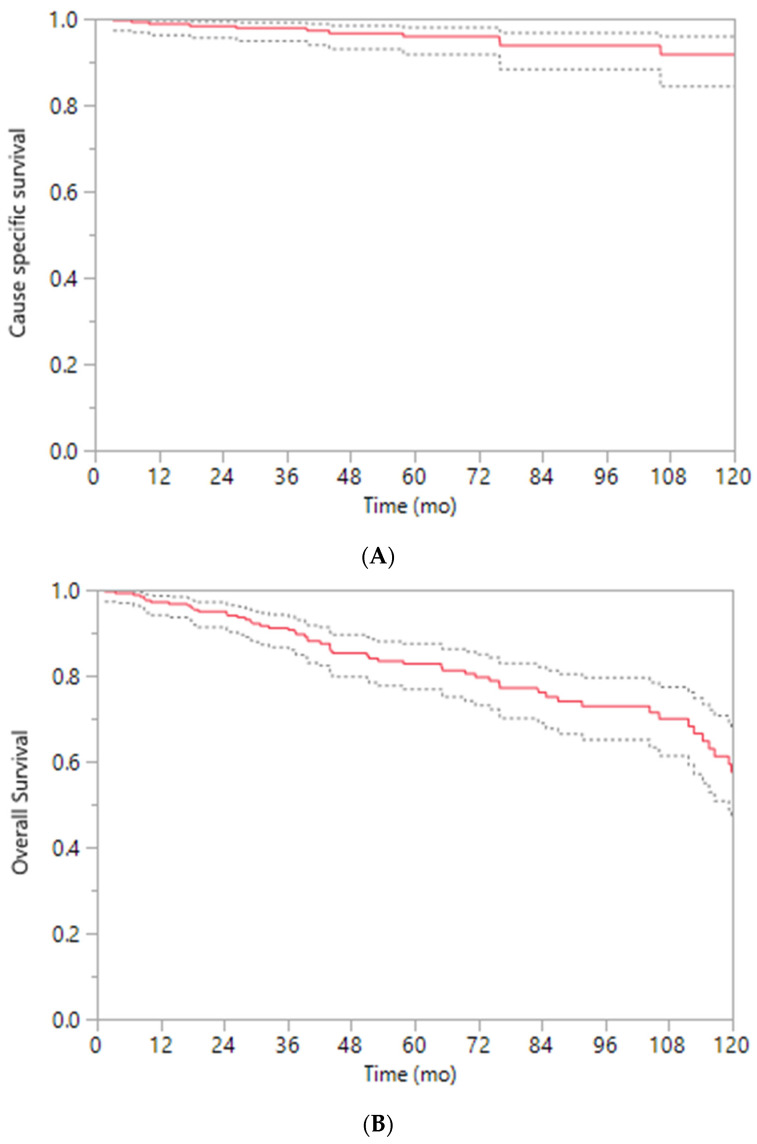
(**A**) **Cause-specific survival** (n = 742). Kaplan-Meier curve (*Y*-axis, % cause-specific survival; *X*-axis, time in months). (**B**) **Overall survival** (n = 742). Kaplan-Meier curve (*Y*-axis, % overall survival; *X*-axis, time in months). Dotted lines represent the 95% confidence interval.

Across NCCN risk groups (low, favorable-intermediate, unfavorable-intermediate, HR, and VHR), 5-year FFBF rates were 91%, 94%, 90%, 83%, and 72%, respectively. Corresponding 5-year FFDM rates were 100%, 98%, 98%, 89%, and 80%, while 10-year CSS was 100%, 100%, 96%, 98%, and 89% (all *p* < 0.01; Table 2). For the overall group, 10-year CSS was 92% (96–84%), and 10-year overall survival was 58% (68–47%) (Figure 1A,B).

Univariate analysis was performed testing covariates of Gleason score, PSA, T stage, race, age, hormonal therapy, and brachytherapy against FFBF and FFDM (Table 3). Gleason score, T stage, and race were all associated with both endpoints (*p* < 0.1), whereas PSA, age, hormonal therapy, and brachytherapy were not. Men with Gleason 9–10 disease had notably worse 5-year FFBF and FFDM (68% and 78%) compared to men with Gleason 8 disease (79% and 88%) and Gleason 7 disease (83% and 87%, respectively, *p* < 0.05).

**Figure 2:**
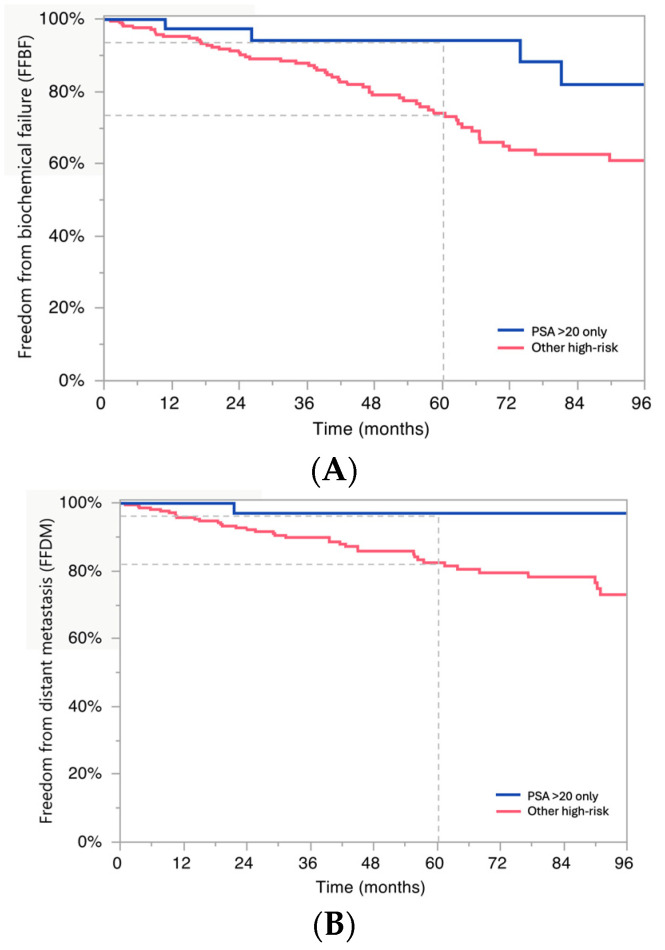
(**A**) **Freedom from biochemical failure** (n = 282). Kaplan-Meier curve (*Y*-axis, % FFBF; *X*-axis, time in months) showing FFBF from radiation treatment completion for HR prostate cancer patients by HR factor (PSA > 20 as the only HR factor, blue, n = 49; all other HR prostate cancer patients, red, n = 233). Dashed-line represents 5-year FFBF for each subgroup. Log-rank test, *p* = 0.05. (**B**) **Freedom from distant metastasis** (n = 282). Kaplan-Meier curve (*Y*-axis, % FFDM; *X*-axis, time in months) showing FFDM from radiation treatment initiation for HR prostate cancer patients by HR factor (PSA > 20 as the only HR factor, blue, n = 49; all other HR prostate cancer patients, red, n = 233). Dashed-line represents 5-year FFDM for each subgroup. Log-rank test, *p* = 0.05.

The median PSA among patients with PSA > 20 as the sole high-risk feature was 30.2 (IQR 23.8–37.1). Men with PSA > 20 as the only high-risk factor had improved 5-year FFBF and 5-year FFDM compared to others with high-risk disease: FFBF: 94% vs. 74%, *p* = 0.05, and FFDM 97% vs. 82%, *p* = 0.05 (Figure 2A,B). These 5-year FFBF and FFDM were not statistically different from outcomes for patients treated with intermediate-risk disease.

In men with PSA > 20 as the sole high-risk factor, 42 men (86%) had Gleason 7 disease, while 7 (14%) had Gleason 6 disease. There was no difference by Gleason score in biochemical outcome (5-year FFBF 100% for Gleason 6 versus 93% for Gleason 7, *p* = 0.28) or distant metastasis (5-year FFDM 100% versus 97%, *p* = 0.68).

A secondary analysis was performed based on the number of NCCN high-risk factors (1, 2, or 3) and demonstrated a correlation with disease outcomes with both 5-year FFBF (83%, 80%, 45%) and 5-year FFDM (89%, 84%, 69%), respectively (all *p* < 0.01) (Table 4).

Additionally, men with Gleason score 9–10 had worse 5-year outcomes compared to those with Gleason ≤ 8 disease (FFBF 68% vs. 82%, *p* = 0.01; FFDM 78% vs. 89%, *p* < 0.01).

### 3.3. Multivariable Predictors of Biochemical Failure

In a multivariable model of HR patients only including covariates with *p* < 0.10 on univariate analysis, Gleason score and T stage were significantly associated with FFBF, and T stage was associated with FFDM, while PSA and race were not associated with either endpoint (Table 5A). Due to the limited sample size of the PSA > 20-only subgroup, interaction testing and propensity score matching were underpowered and not performed. A multivariable model of all patients similarly showed that PSA and race were not associated with FFBF or FFDM (Table 5B).

## 4. Discussion

Risk stratification within the traditional NCCN ‘high risk’ classification system defined by the presence of a single risk factor of PSA > 20, clinical stage T3–4, or Gleason score 8–10 has been studied previously. Several studies have attempted to define subcategories of risk [6,18,19,20,21] to individualize therapy.

A SEER database analysis of 151,799 men defined seven risk groups, among which the best survival was observed for men with a single high-risk factor of T3 or PSA > 20, and the worst survival was observed for men with all three risk factors [18]. A large single institutional analysis of 3618 men observed favorable survival for those with T1c, Gleason 8, and PSA < 10, or T1c, Gleason 6, and PSA > 20 disease (“favorable high-risk”). These two favorable subgroups were validated in a 13,275 SEER-Medicare patient cohort by showing that their 5-year cancer mortality-risk of 1.3% was similar to unfavorable intermediate risk disease [19]. This “favorable high-risk” classification was validated in a prospective cohort from the PLCO Cancer Screening trial, showing comparable cancer-specific mortality to those with unfavorable intermediate-risk disease (2.2% at 8 years for both categories) [6]. Using the Decipher genomic classifier, Muralidhar et al. evaluated a 3-tiered risk classification system for ‘high-risk’ disease and demonstrated that the “favorable high risk” group had the lowest proportion of patients classified as high risk by Decipher (50.4%) compared to “standard high risk” (cT3a, Gleason 4 + 4 and PSA > 10, and Gleason 7 and >20 PSA) and “very high risk” (cT3b+, Primary Gleason 5), 64.2% and 81.6%, respectively [20]. Finally, using the SEER database, Muralidhar et al. showed that external beam radiotherapy plus brachytherapy boost is associated with improved cancer-specific mortality only in men with non-“favorable high risk” (5.3% vs. 3.9% at 5 years) [21].

While these data provide valuable insight regarding the biological and prognostic heterogeneity of ‘high risk’ patients, there are several limitations to consider. First, the SEER analysis by Song et al. [18] and the PLCO analysis by Butler et al. [6] included patients treated with external beam radiotherapy between 2004–2015 and 1993–2001, respectively. Radiotherapy details were not provided, and it is unclear what proportion of patients received dose-escalated radiotherapy, which would be considered standard of care today. Second, the studies above do not report biochemical control or distant metastases rates, which are important endpoints for patients and physicians as they influence subsequent patient therapy. Finally, among patients with >20 PSA, Muralidhar et al. [19,20] only considered patients as “favorable high risk” if the Gleason score was 6; thus, it is unclear how patients with Gleason 7 and >20 PSA fare.

In the present study, men with high-risk prostate cancer whose only high-risk feature was a PSA level > 20 ng/mL demonstrated superior 5-year FFBF and FFDM compared to other high-risk patients, and their outcomes were comparable to patients treated with intermediate-risk disease. In contrast, individuals with Gleason scores of 8–10 experienced significantly worse FFBF and FFDM rates compared to those with lower Gleason scores. In this cohort, PSA was not as strongly associated with outcome and appeared to be the weakest prognostic factor among the high-risk features evaluated. These findings support the rationale to sub-stratify the high-risk group to personalize treatment with RT for men with prostate cancer.

A notable finding is that patients with PSA > 20 as their sole high-risk factor had improved outcomes despite receiving less aggressive treatment with radiation, including less pelvic nodal RT and ADT duration. This corroborates an earlier multi-institutional report [7] and suggests a possible benefit of reclassifying these men into a lower-risk disease category. A potential explanation for why an isolated elevation in PSA may not correlate with worse clinical outcomes could in part be due to the tumor location. Prostate cancers arising in the transitional zone have been shown to produce disproportionately higher PSA levels compared to those originating in the peripheral zone—the site where most clinically significant cancer develops [22,23]. In such cases, elevated PSA may reflect increased PSA production rather than aggressive tumor biology. This variability in PSA expression based on tumor location may contribute to an overestimation of disease severity, thus diminishing the prognostic utility of PSA when considered in isolation. Given that the tumor location within the prostate gland was not known, this reasoning is a speculative hypothesis supported by prior literature but not able to be concluded from our data.

In contrast, it has been shown that high-risk patients with a low PSA value but higher-grade disease are at greater risk of death. These patients are more likely to have neuroendocrine genomic features, which may have a poorer response to ADT [24]. Building on this, studies have shown significantly worse outcomes in patients with Gleason 9–10 disease compared to Gleason 8, with Gleason 9–10 disease having less survival benefit from ADT [1]. Our study did demonstrate inferior outcomes in both FFBF and FFDM for men with Gleason 9–10 compared to Gleason 8 disease on univariate analysis.

A shorter course of ADT may be appropriate for certain HR patients. It has been previously shown that short-term ADT in conjunction with dose-escalated RT may be appropriate for individuals with high-risk, localized disease who have clinical T1–T2 prostate cancer [7,25]. In addition, the use of predictive genomic tests such as Decipher may help in the decision to intensify or de-intensify treatment. In a study by Spratt et al., patients categorized as high risk by traditional clinicopathologic factors had a 10-year cumulative incidence of metastases ranging from 5.5% in Decipher low-risk patients to 26.7% in Decipher high-risk patients [26]. Prospective validation of these findings is essential. Ongoing trials such as NRG-GU009 (PREDICT-RT), which risk-stratifies high-risk prostate cancer using genomic classifiers to guide treatment intensification or de-intensification, may provide an ideal framework to test whether patients with isolated PSA elevation derive limited benefit from prolonged systemic therapy. Similarly, ArteraAI, an AI-driven prognostic tool, could provide information on which high-risk patients actually benefit from the addition of longer-term ADT [27]. Integration of these tools may help distinguish patients with biologically indolent disease despite high PSA from those requiring treatment intensification. Given the lack of prospective comparative data showing equivalent outcomes for short-term ADT in high-risk prostate cancer treated with RT, our recommendation would still be to treat high-risk men with RT and long-term ADT, although a de-escalated approach may be appropriate in certain men with comorbidities and lowered life expectancy. Less aggressive approaches, such as with the replacement of long-term LHRH agonists with 5-alpha reductase inhibitors, have been shown to yield similarly high rates of disease control with less disruption to hormonal quality of life [28]. The optimal treatment decision for men with prostate cancer is one that balances benefit and risk while acknowledging the individualized contribution of clinical risk factors, medical comorbidity and life expectancy, and patient preference.

Several limitations merit consideration. First, the retrospective, single-institution design introduces unavoidable selection bias and the potential for unmeasured confounders, including clinician-driven differences. Notably, patients with PSA > 20 as their sole high-risk factor had differences in treatment intensity regarding coverage of pelvic lymph nodes and duration of ADT. This may have been influenced in part by clinician perception of lower biologic risk or by patient medical comorbidity. While these treatment differences could confound outcome comparisons, the relatively favorable outcome in the PSA > 20 high-risk group was observed despite a lower treatment intensity for these men. Propensity score matching was considered but not feasible without substantial loss of statistical power given the modest size of this subgroup. Second, the median follow-up of 53 months is reasonable to analyze biochemical recurrence; it is limited in the assessment of late endpoints, including metastasis-free survival and cancer-specific mortality. Biochemical recurrence is clinically meaningful, as it can prompt discussion of salvage therapies, but prostate cancer is characterized by a prolonged natural history, and longer follow-up is required to determine whether the favorable early outcomes observed in PSA-only high-risk men persist over time. Similarly, toxicity data, including genitourinary, gastrointestinal, and long-term ADT-related effects, were not included in this analysis, limiting assessment of treatment trade-offs. It could be expected that shorter-term ADT would mitigate the risks of longer-term hormonal deprivation [29,30], but this has not been proven in this cohort. Third, there was some heterogeneity in treatment methods used over the long study interval. Radiation techniques evolved from 3-dimensional conformal RT to intensity-modulated RT, and modern molecular imaging such as PSMA PET was not routinely available, potentially leading to understaging in some patients. Additionally, Gleason grading predates contemporary ISUP refinements and is subject to the possibility of grade migration [31]. Finally, an important consideration in this analysis is the racial composition of the PSA > 20 ng/mL subgroup, which included a higher proportion of Black men compared with the overall cohort. Prior studies have demonstrated that Black men treated with definitive radiation therapy may experience equivalent or superior oncologic outcomes compared with White men when access to care is equitable [32], despite presenting with higher PSA levels and more advanced disease features. Similarly, differential responses to androgen deprivation therapy have been reported [33]. While race was not independently associated with biochemical or metastatic outcomes in our univariate analyses or multivariable models, our modest sample size could limit definitive conclusions. These findings underscore the need for multi-institutional validation in racially diverse cohorts and caution against extrapolation without further study. Future studies could stratify outcomes by race and include genomic or molecular correlates to better understand varying treatment effects. While our findings suggest that PSA > 20 as an isolated high-risk feature is associated with favorable outcomes, this conclusion should be interpreted as hypothesis-generating rather than practice-changing. Long-term ADT in combination with definitive RT remains the standard of care for high-risk prostate cancer based on randomized evidence. Any consideration of treatment de-escalation should occur only in carefully selected patients through shared decision-making or within prospective clinical trials. Future studies integrating genomic classifiers such as Decipher or artificial intelligence-based tools like ArteraAI may help distinguish patients who derive limited benefit from prolonged systemic therapy from those who require treatment intensification. Prospective validation in multi-institutional cohorts with contemporary imaging and longer follow-up will be critical to refine risk stratification and personalize therapy for men currently classified as high risk based on PSA alone.

## 5. Conclusions

Select patients with PSA > 20 ng/mL but otherwise favorable clinical characteristics (cT1–2, Gleason ≤ 7) demonstrate excellent outcomes following definitive RT. These findings support further refinement of high-risk classification and underscore the need for prospective studies to evaluate individualized treatment intensity in this subgroup.

## Figures and Tables

**Table 1:** **Demographic and clinical characteristics of patients.** NCCN High Risk, PSA > 20 only includes patients with PSA > 20 as the sole high-risk factor. NCCN High Risk, other, includes all Other patients with either multiple high-risk factors or clinical T3 or Gleason 8–10 as a sole high-risk factor. ^1^ NCCN = National Comprehensive Cancer Network, ^2^ PSA = Prostate Specific Antigen, ^3^ Gy = Gray, ^4^ ADT = Androgen Deprivation Therapy.

	All Patients (n = 282)	NCCN ^1^ High Risk, PSA > 20 Only (n = 49)	NCCN ^1^ High Risk, Other (n = 233)	*p*
**Age, median (years) (IQR)**	68 (63–74)	67 (63–72)	69 (63–75)	0.51
**Race**				
White	87 (31%)	6 (13%)	81 (35%)	
Black	185 (66%)	42 (88%)	143 (62%)	0.006
Asian	5 (2%)	0	5 (2%)	
Hispanic	1 (0%)	0	1 (0%)	
Other	1 (0%)	0	1 (0%)	
**T stage**		1 (2%)	2 (1%)	<0.0001
Tx	3 (1%)	32 (65%)	89 (38%	
T1–T2a	121 (43%)	15 (31%)	28 (12)	
T2b–T2c	43 (15%)	1 (2%)	114 (9%)	
T3–4	115 (41%)			
**Gleason Score,**				<0.0001
6	12 (4%)	7 (14%)	5 (2%)	
7	74 (26%)	42 (86%)	32 (14%)	
8	99 (35%)	0	99 (42%)	
9	91 (32%)	0	91 (39%)	
10	6 (2%)	0	6 (3%)	
**Median PSA ^2^ (ng/mL), (IQR)**	19 (9.5–37.7)	30.2 (23.8–37.1)	13.2 (7.9–37.8)	0.78
**Median dose (Gy ^3^)**	78	78	79.2	0.67
**Pelvic nodal radiation**	161 (57%)	20 (41%)	141 (61%)	<0.01
**Brachytherapy boost**	19 (6%)	2 (4%)	17 (7%)	0.39
**ADT ^4^ administered**	262 (94%)	41 (84%)	221 (96%	0.03
Median time (IQR) (months)	21 (19–28)	10 (6–21)	24 (11–28)	
**Follow up, median (IQR) (months)**	53 (25–88)	41 (18–81)	54 (30–90)	0.09

**Table 2:** **Primary outcomes (n = 742).** Five-year FFBF, 5-year FFDM, and 10-year CSS outcomes by prostate cancer risk group with 95% confidence intervals. ^1^ FFBF: Freedom from biochemical failure, ^2^ FFDM: Freedom from distant metastasis, ^3^ CSS: Cancer-specific survival. 95% confidence intervals are shown in parentheses.

Risk Group	5-Year FFBF ^1^ (%)	5-Year FFDM ^2^ (%)	10-Year CSS ^3^ (%)	*p* (All)
Low (n = 107)	91 (96–83)	100	100	<0.0001
Favorable Intermediate (n = 103)	94 (98–86)	98 (100–89)	100	
Unfavorable Intermediate (n = 220)	90 (94–84)	98 (99–83)	95 (99–78)	
High Risk (n = 146)	83 (89–73)	89 (94–80)	92 (98–75)	
Very High Risk (n = 136)	72 (80–62)	80 (87–71)	90 (95–80)	

**Table 3:** Univariate analysis of clinical variables for 5-year freedom from biochemical failure (FFBF) and freedom from distant metastasis (FFDM).

Variable	5-Year FFBF	*p*	5-Year FFDM	*p*
**Gleason score**				
6	100%	0.0381	100%	0.0298
7	83%		87%	
8	79%		88%	
9–10	68%		78%	
**PSA**				
>20 vs. <20	75% vs. 77%	0.1714	85% vs. 82%	0.9801
**T-stage**				
T1–T2a	86%	0.0001	90%	0.0004
T2b–T2c	86%		93%	
T3–4	62%		74%	
**Race**				
Black	83%	0.0212	78%	0.0776
White/other	68%		89%	
**Age**				
>68 vs. <68	71% vs. 79%	0.2105	82% vs. 87%	0.5250
**Hormonal therapy**	77% vs. 78%	0.8561	85% vs. 83%	0.9924
Yes vs. No				
**Brachytherapy**	90% vs. 76%	0.1138	100% vs. 84%	0.1143
Yes vs. No				

**Table 4:** **Primary outcomes.** Five-year FFBF, 5-year FFDM, and 10-year CSS outcomes by prostate cancer risk group. 1 FFBF: Freedom from biochemical failure, 2 FFDM: Freedom from distant metastasis, 3 CSS: Cancer-specific survival. 95% confidence intervals are shown in parentheses.

Number of High-Risk Factors	n (%)	5-Year FFBF (%)	5-Year FFDM (%)	10-Year CSS (%)
1	157 (56%)	83% (89–75)	89% (94–81)	94% (98–80)
2	84 (30%)	80% (89–68)	84% (91–72)	88% (96–72)
3	41 (15%)	45% (64–27)	69% (83–51)	90% (97–72)
*p* value		<0.01	<0.01	0.29

**Table 5:** (**A**) Multivariable analysis of Freedom From Biochemical Failure (FFBF) and Freedom from Distant Metastasis (FFDM) in men with high-risk prostate cancer (n = 282). (**B**) Multivariable analysis of Freedom From Biochemical Failure (FFBF) and Freedom from Distant Metastasis (FFDM) in all men with prostate cancer (n = 742).

(**A**)				
	**FFBF**		**FFDM**	
	**Risk Ratio**	***p***	**Risk Ratio**	***p***
T Stage T2b+ vs. T1c–2a	2.51 (1.33–4.72)	0.0024	2.24 (1.02–4.91)	0.0439
Gleason score		0.0551		0.4126
Gleason 8 vs. 7	1.25 (0.63–2.49)		0.84 (0.38–2.13)	
Gleason 9–10 vs. 7	1.52 (0.80–2.86)		1.60 (0.74–3.50)	
Race Black vs. White/other	0.75 (0.44–1.28)	0.2961	0.77 (0.39–1.50)	0.4373
PSA (continuous)	1.00 (0.99–1.00)	0.6320	1.00 (1.00–1.00)	0.6963
(**B**)				
	**FFBF**		**FFDM**	
	**Risk Ratio**	***p***	**Risk Ratio**	***p***
T Stage T2b+ vs. T1c–2a	2.32 (1.53–3.53)	<0.001	3.43 (1.76–6.69)	0.003
Gleason score		0.0238		0.0532
Gleason 8 vs. 7	1.71 (1.10–2.66)		2.21 (1.13–4.19)	
Gleason 9–10 vs. 7	2.09 (1.11–3.92)		NA (NA)	
Race Black vs. White/other	0.88 (0.59–1.31)	0.5435	0.72 (0.40–1.31)	0.2803
PSA (continuous)	1.00 (1.00–1.00)	0.5287	1.00 (1.00–1.00)	0.5136

## Data Availability

To ensure compliance with the existing IRB approval letter and HIPAA compliance for patients, the full de-identified dataset cannot be made available without prior written approval from the University of Chicago Hospital IRB. Requests for de-identified data can be made to the corresponding author.

## Abbreviations

NCCN	National Comprehensive Cancer Network
ADT	Androgen Deprivation Therapy
FFBF	Freedom From Biochemical Failure
FFDM	Freedom From Distant Metastasis
PSA	Prostate Specific Antigen
HR	High Risk
VHR	Very High Risk
IMRT	Intensity Modulated Radiation Therapy
GnRH	Gonadotropin Releasing Hormone
RT	Radiation Therapy
